# Effects of Edentulism on Mandibular Morphology: Evaluation of Panoramic Radiographs

**DOI:** 10.1155/2014/254932

**Published:** 2014-08-18

**Authors:** Rıdvan Okşayan, Bayram Asarkaya, Nizamettin Palta, İrfan Şimşek, Oral Sökücü, Eren İşman

**Affiliations:** ^1^Department of Orthodontics, Faculty of Dentistry, Osmangazi University, 26000 Eskişehir, Turkey; ^2^Department of Orthodontics, Faculty of Dentistry, Gaziantep University, 27310 Gaziantep, Turkey; ^3^Department of Prosthodontics, Faculty of Dentistry, Gaziantep University, 27310 Gaziantep, Turkey

## Abstract

*Purpose*. The objective of this study was to evaluate morphological changes of the mandible in edentulous and dentate subjects using panoramic radiographs. *Materials and Methods*. Panoramic radiographs were divided into the following three groups: Group I (completely edentulous group), Group II (old dentate group) and Group III (young dentate group). The research parameters of gonial angle, condylar height, ramus height, ramus notch depth, and antegonial notch depth were measured on panoramic radiographs. The Kruskal-Wallis statistical test was used to determine significant differences in mandibular morphological parameters among the three groups. The Mann-Whitney *U* test was used to compare the subgroups. *Results*. Significant differences were found only in ramus height between three groups. According to the Mann-Whitney *U* test, a significant difference was exhibited among Groups I and II in the ramus height parameter. No significant differences were found in the gonial angle, condylar height, ramus notch depth, or antegonial notch depth when comparing the young dentate, old dentate, and completely edentulous subjects. *Conclusions*. Significant differences were found only in ramus height between the groups. Ramus height may be an indicator that changed by years and tooth loss. It must be considered that ramus height can be decreased in edentulism.

## 1. Introduction

Some problems such as dental pain, periodontal disease, dental caries, and expensive dental therapies can be solved with tooth extraction. These therapies may cause edentulism, which unfortunately may represent the beginning of various new problems. Edentulism can essentially influence general and oral health and at the same time affect overall quality of life [1]. With complete dentures, speech, chewing, and taste of food cannot be fully guaranteed [2]. Depending on age, gender, and dentoalveolar condition of patients, many morphological and anatomical changes are exhibited in edentulous mandibles [3, 4]. In edentulism, changes in the mandibular bone after tooth loss may cause residual ridge resorption [5]. Several studies have focused on mandibular morphological and anatomical changes between edentulous and dentate subjects [6–8]. Generally, previous studies have used gonial angle (GA), antegonial notch depth (AND), ramus notch depth (RND), condylar height (CH), and ramus height (RH) to evaluate the anatomical changes in edentulous subjects [5, 6, 9, 10].

Panoramic radiographs have wide, complementary clinical radiological applications in dentistry. Alhaija stated that panoramic radiographs are a useful tool for the measurement of GA [5, 11]. According to the mandibular morphology, GA dimensions can change with age, tooth loss, and denture use [12]. GA size has shown to be related to the development of masticatory muscles [13]. The resulting upward curving of the inferior border of the mandible anterior to the angular process (gonion) is known as the antegonial notch [10]. Some studies have investigated the possibility that antegonial notch morphology predicts mandibular growth [14]. It is possible that RND may indicate the occurrence of previous condylar bone change [10]. Mangla et al. stated that RH was significantly bigger in the hypodivergent group than in the hyperdivergent group and males have greater RHs than females [15]. The purpose of this study was to compare the mandibular morphological measurements of totally edentulous, young dentate, and old dentate subjects.

## 2. Materials and Methods

### 2.1. Study Sample

Panoramic radiographs had been selected from Gaziantep University Dentistry Faculty Orthodontic and Prosthodontic Departments Archives. Seventy-two panoramic radiographs of the following three groups of subjects were evaluated: Group I (completely edentulous group), 24 subjects (mean age 69.7 years old; all older than 50 years); Group II, (old dentate group), 24 subjects (mean age 62.2 years old; all older than 50 years); Group III (young dentate group), 24 subjects (mean age 18.8 years old; range, 16–21 years old) (Table 1). GA, AND, RND, RH, and CH were measured on panoramic radiographs. In edentulous group, all subjects were denture wearers. In old and young dentate groups, all the teeth were present except second and third molars. Also, in dentate groups, there were no partial denture wearers. Patients with craniofacial syndromes and facial traumas were excluded from the study samples in both groups.

### 2.2. Radiographic Evaluation

**Figure 1:**
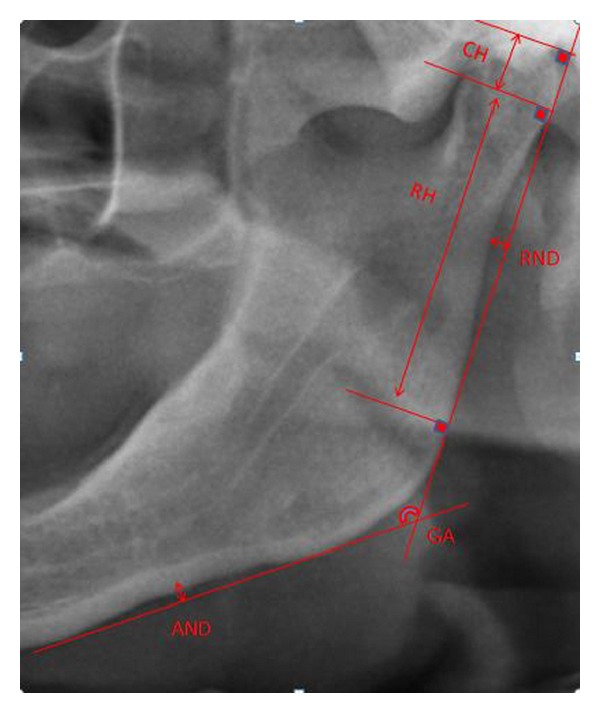
Measuring method used for the gonial angle, ramus height, condylar height, antegonial notch depth, and ramal notch depth on panoramic radiograph.

The radiographs were taken with the same digital machine (Sirona, XG 3, Munchen, Germany). The criteria for selection of patients radiographs were that they had to be of high quality and sharpness, and all radiographs had to be taken using the same apparatus by the same technician. In addition to these criteria, the condylar area, the posterior border of the ramus, and the lower border of the mandible had to be clearly readable on the left part of panoramic radiograph to define skeletal landmarks. Each panoramic radiograph was traced on acetate with a 0.5 mm mechanical pencil and by the same investigator (B.A.) as follows (Figure 1). Ten randomly selected radiographs were traced by the same investigator again and Pearson correlation coefficient was between 0.80 and 0.91 for all the variables.

1. Gonial Angle (GA): it was traced between the imaginary tangential line along the posterior border of the mandibular ramus and the inferior border of the mandible [6];
2. Condylar Height (CH): the line was drawn perpendicular to the ramus tangent line at the level of the most lateral image of the condyle. Another line was drawn perpendicular to the ramus tangent line at the level of the most superior image of the condyle. CH was the perpendicular distance between the lines [6];
3. Ramus Height (RH): the line was drawn perpendicular to the ramus tangent line at the level of the most lateral image of the ramus. RH was the distance between the lines [6];
4. Antegonial Notch Depth (AND): it was measured as the distance along a perpendicular line from the deepest point of the antegonial notch concavity to a line parallel to the inferior cortical border of the mandible [10];
5. Ramal Notch Depth (RND): it was defined as the distance along a perpendicular line from the deepest point of the ramus notch concavity [10].

### 2.3. Statistical Analysis

Statistical analysis was performed with SPSS statistics for Windows Version 16 (SPSS, Chicago, IL, USA). Descriptive statistics, including the mean, standard deviation, median, and minimum and maximum values, were calculated for each of the three groups (Table 1). Kolmogorov-Smirnov and Shapiro-Wilk statistical tests were conducted to assess the normality of variances (*P* < 0.05). The Kruskal-Wallis nonparametric statistical test was used to determine significant differences between the three groups, while the Mann-Whitney* U* test was used to compare the subgroups.

## 3. Results

Significant differences were found only in RH among the three groups (*P* < 0.05) (Table 2). In the comparison of subgroups according to the Mann-Whitney* U* test, a significant difference was found between Groups I and II in the ramus height parameter (*P* < 0.05) (Table 3). Edentulous subjects showed lower mean RH values than the old dentate group. According to the Kruskal-Wallis statistical test, no significant differences were found between GA, CH, RND, or AND when comparing young dentate, older dentate, and completely edentulous subjects (*P* > 0.05). There were no significant differences in GA among the groups. However, edentulous subjects showed higher mean values of GA than young and old dentate groups. Edentulous subjects showed higher mean values of CH than dentate subjects, but there was no significant difference between the three groups. The young dentate group demonstrated lower mean values of RND and AND, but there were no significant differences between groups.

## 4. Discussion

In this study, five mandibular morphological and anatomical parameters, including one angular and four linear ones, were measured [7, 8, 10, 16]. The panoramic radiograph has been used to measure the parameters in previous studies [6], as a panoramic radiograph could prevent the superimposition of images, which is seen in lateral cephalograms; from this perspective, they are regarded to be unsurpassed to cephalograms [5]. Additionally, Okşayan et al. revealed that orthopantomograms can be used as an alternative radiographic technique to evaluate gonial angle in orthodontic patients [17].

Many studies have demonstrated that the GA widens in edentulous subjects; conversely, researchers have reported nonwidening of the GA in edentulous subjects [6, 7, 16]. Ohm and Silness reported that GA becomes larger with aging and advancing edentulism [4]. In contrast, Xie and Ainamo found that the GA did not change with age, gender, or dental status. Mostly, dentate subjects have a smaller GA than edentulous subjects [16]. Ceylan et al. found that there was no significant difference in GA between the dentulous and edentulous states [7]; these findings support our study results concerning GA. According to our study, there were no significant differences in GA between young dentate, old dentate, and completely edentulous groups. However, edentulous subjects showed higher mean values of GA. In partial or total edentulism, function of the masseter and temporal muscle is decreased and this may affect the mandibular angle [5]. In addition to this, Keen also stated that the dentures of edentulous patients prevented widening of the mandibular angle [18]. This difference between studies may have resulted from using of different radiographs, such as panoramic and cephalometric ones. In addition, gender mismatch may have affected this difference.

No significant difference was found in CH between groups. However, the completely edentulous group showed higher mean values of CH. According to this result, we can say that there was no relationship between dentition and CH. This condition may have occurred because condylar height measurement in panoramic radiographs is controversial. Türp et al. studied the asymmetry of the condylar area and ramus heights on panoramic radiographs; after comparison with direct measurements from skulls, they found a low correlation between measurements [19]. Joo et al. found a negative correlation between GA and CH in both sexes [6], and these findings coincided with the results of our study.

Depending on age, sex, and dental condition of patients, mandibles with edentulism show many morphological and anatomical differences. According to our results, RH values showed significant differences between groups (*P* < 0.05). Old dentate subjects showed higher mean values of RH than those in the edentulous group. Subtelny reported that decreased ramal height correlated with reduced lower jaw development, and this condition may cause degenerative mandibular condyle and asymmetry [20]. According to Mangla et al., RH was significantly smaller in the hyperdivergent group than in the hypodivergent group, and gender resulted in a significant difference, with females having smaller RHs than males [15]. Edentulous subjects showed decreased mean values of RH results in our study, and this condition may cause GA values to become larger in edentulous subjects. The decrease in RH is a result of decompensation in alveolar ridge loss.

AND was affected by many factors, including muscle functions, osteoporotic and osteopenic conditions, and mandibular growth rotation. Dutra et al. reported that AND was significantly decreased in subjects with normal bone mass compared to osteopenic and osteoporotic patients [8]. Singer et al. notified that subjects with deep antegonial notches had a more retrusive lower jaw, smaller ramus height, and higher gonial angle than individuals with a shallow notch depth [21]. Some studies have reported that orthodontists might predict mandibular growth according to antegonial notch depth [22, 23]. Mangla et al. reported that greater antegonial notch depth values and no sexual dimorphism were found between hyperdivergent and hypodivergent subjects [15]. In this study, AND values showed no significant difference between groups. Keen also observed that the dentures of edentulous patients prevented widening of the mandibular angle [18]. This may also have affected our study results; in addition to this, AND may be affected by mandibular angle change.

According to our results, there were no significant results between the young dentate group, old dentate group, and completely edentulous group. RND measurement was identified as the distance along a perpendicular line from the deepest point of the ramus notch concavity [10]. Ali et al. reported that there was a relationship between condylar bone change and ramus notch depth; they found that increased ramus notch depth might be related to condylar posterior bone apposition [10]. In our study, the young dentate group showed lower mean values in RND; consequently, it may be said that RND increases with age.

This study has limitations, such as the predicted growth pattern of the mandible, gender mismatch, and skeletal classifications of the participants. However, this study is important in the fact that it shows the effects of different dental states and age conditions on mandibular morphology.

## 5. Conclusion

In conclusion, RH was affected by edentulism, although the only significant difference was found between the edentulous and old dentate groups. Edentulism may have a potential to decrease RH. According to the results, edentulism may affect GA, AND, and RND, and the deepness of notches may become larger with age. The comparison of different ages allow us to predict the mandibular morphological parameters before edentulous state.

## Figures and Tables

**Table 1:** Descriptive statistics (subject number, sex, and mean age) of study groups.

Groups	Number of subjects (*n*)	Sex (female /male)	Mean age
I	24	13/11	69.70 ± 8.10
II	24	17/7	62.20 ± 5.40
III	24	15/9	18.83 ± 1.40

**Table 2:** Comparison of variables with Kruskal-Wallis test related to groups.

Variables	Group I mean ± SD	Group II mean ± SD	Group III mean ± SD	*P* value
Gonial Angle (°)	124.12 ± 7.75	120.39 ± 6.79	121.95 ± 6.69	0.182
Condylar Height (mm)	9.81 ± 1.94	7.64 ± 1.41	8.36 ± 1.60	0.186
Ramus Height (mm)	48.95 ± 6.37	53.17 ± 6.04	50.08 ± 5.87	0.041*
Antegonial Notch Depth (mm)	2.12 ± 1.36	2.32 ± 2.08	1.51 ± 1.12	0.196
Ramal Notch Depth (mm)	3.72 ± 1.15	3.97 ± 1.22	3.30 ± 1.09	0.137

*P < 0.05: statistically significant.

**Table 3:** Mean ramus height values, standard deviations, and Mann-Whitney  *U*  test grouping.

Variable	Number of subjects (*n*)	Groups	Mean (Sd) values	Mann-Whitney *U* grouping
	24	I	48.95 (±6.37)	A
Ramus Height	24	II	53.17 (±6.04)	B
	24	III	50.08 (±5.87)	AB

Different capital letters indicate that ramus height values are significantly different at *P* < 0.05.
